# Therapeutic efficacy of recombinant human growth hormone in children with different etiologies of dwarfism from a pharmacoeconomic point of view

**DOI:** 10.1097/MD.0000000000038350

**Published:** 2024-06-21

**Authors:** Yanxia Ma, Jianping Sheng, Lijie Wang, Yanan Zhang, Lin Liu

**Affiliations:** a School of Clinical Medicine, Weifang Medical University, Weifang, China; b School of Anesthesiology, Weifang Medical University, Weifang, China; c Department of Endocrinology, Weifang People’s Hospital, Weifang, China.

**Keywords:** growth hormone, growth hormone deficiency, idiopathic short stature

## Abstract

Treatment outcomes for different causes of childhood dwarfism vary widely, and there are no studies on the economic burden of treatment in relation to outcomes. This paper compared the efficacy and healthcare costs per unit height of recombinant human growth hormone (rhGH) for the treatment of growth hormone deficiency (GHD) and idiopathic short stature (ISS) with a view to providing a more cost-effective treatment option for children. We retrospectively analyzed 117 cases (66 cases of GHD and 51 cases of ISS) of short-stature children who first visited Weifang People’s Hospital between 2019.1 and 2022.1 and were treated with rhGH for 1 to 3 years to track the treatment effect and statistically analyzed by using paired *t* tests, non-parametric tests, and chi-square tests, to evaluate the efficacy of rhGH treatment for GHD and ISS children and the medicinal cost. The annual growth velocity (GV) of children with GHD and ISS increased the fastest during 3 to 6 months after treatment and then gradually slowed down. The GV of the GHD group was higher than that of the ISS group from 0 to 36 months after treatment (*P* < .05 at 3, 6, 9, and 12 months); the height standard deviation scores (HtSDS) of the children in the GHD and ISS groups increased gradually with the increase of the treatment time, and the changes in the height standard deviation scores (ΔHtSDS) of the GHD group were more significant than those of the ISS group (*P *< .05 at 3, 6, 9, and 12 months). (2) The medical costs in the pubertal group for a 1-cm increase in height were higher than those of children in the pre-pubertal group at the same stage (3 to 24 months *P *< .05). The longer the treatment time within the same group, the higher the medical cost of increasing 1cm height. RhGH is effective in treating children with dwarfism to promote height growth, and the effect on children with GHD is better than that of children with ISS; the earlier the treatment time, the lower the medical cost and the higher the comprehensive benefit.

## 1. Introduction

In recent years, more and more parents have been concerned about their children’s growth and development, and a study has shown that short stature seriously affects the physical and mental health of the affected children. Parents and children themselves have an urgent need for treatment of short stature. The primary goal of recombinant human growth hormone (rhGH) treatment in children is to increase and normalize growth rates (GV) to achieve adequate adult heights.^[1,2]^ Due to the expensive cost of growth hormone therapy, the economic burden of rhGH treatment for dwarfism concerns many patients’ families. According to relevant studies,^[3]^ the causes of short stature are complex, and the common causes are growth hormone deficiency (GHD) and idiopathic short stature (ISS). The purpose of this study is to analyze the efficacy of growth hormone in improving the height of children with dwarfism by collecting children with idiopathic dwarfism and growth hormone deficiency at different ages, exploring the clinical data before and after rhGH injection, comparing the medical costs of rhGH treatment for children with growth hormone deficiency and idiopathic dwarfism at different ages and in different periods of treatment, and improving the effectiveness of the clinical treatments with the hope of providing more favorable treatment for the children in an economic perspective. To enhance the effectiveness of clinical medicine, we hope to provide better diagnosis and treatment programs for children from a financial point of view.

## 2. Objects and methods

### 2.1. Objects of study

A total of 117 children diagnosed with dwarfism and treated with rhGH for more than one year in the outpatient clinic of the Department of Endocrinology of Weifang People’s Hospital between January 2019 and January 2022 were collected and categorized into the GHD group and ISS group. The development of secondary sexual characteristics was based on the B2 stage of average breast growth and testicular volume over 4 ml as the criteria for entering puberty.

#### 2.1.1. Inclusion criteria

**Figure 1.**
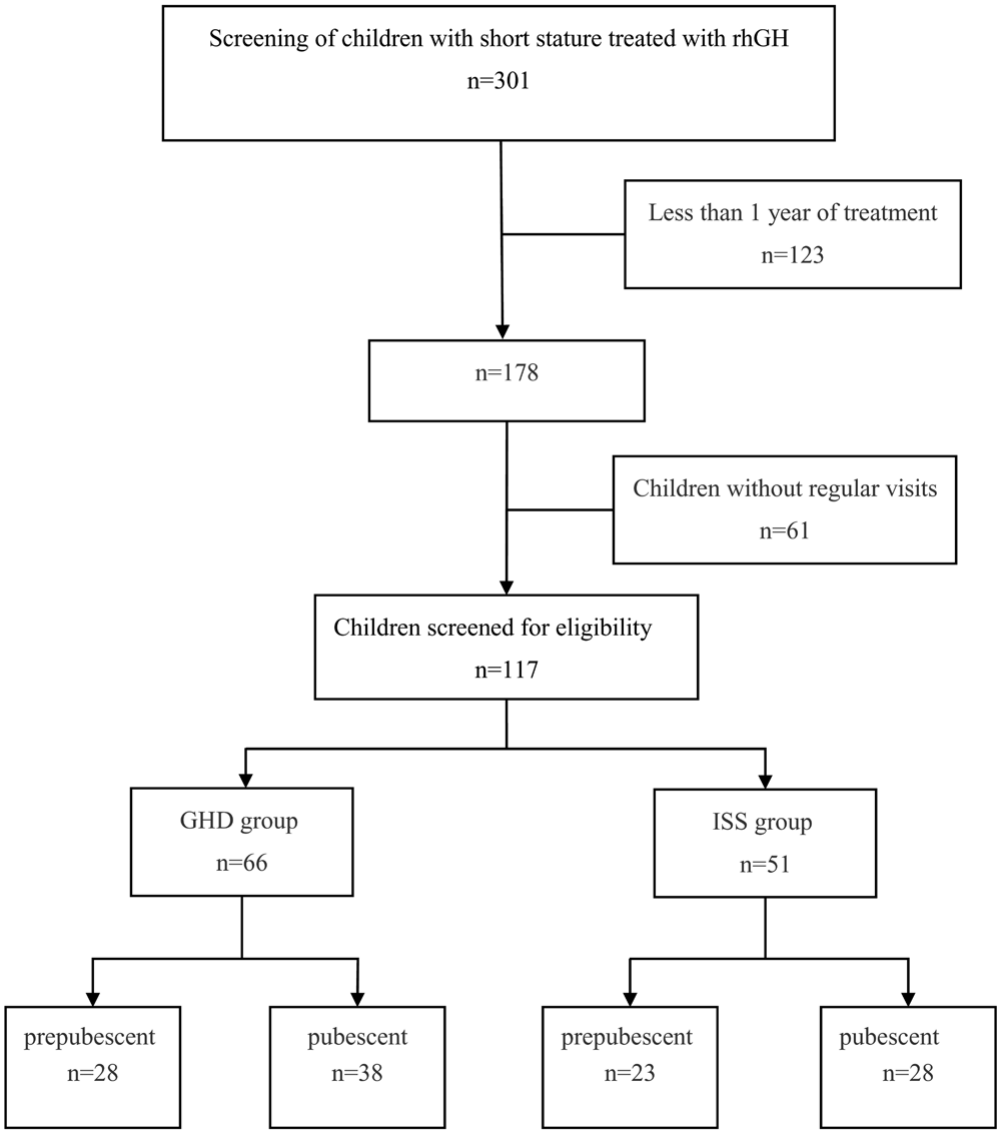
Flow diagram of included and excluded trials.

The diagnosis and rhGH treatment of ISS and GHD refer to the professional guidelines.^[4]^ Flow diagram of included and excluded trials (Fig. 1).

GHD group:

1. The height of normal children of the same race, age and sex is lower than the 3rd percentile (<P3) or less than two standard deviation score.
2. The results of growth hormone stimulation test of the two drugs indicated that the peak value of GH was low (<10 ng/mL).
3. delayed bone age.

ISS group:

1. The height of normal children of the same race, age and sex is lower than the 3rd percentile or less than two standard deviation score.
2. Growth hormone stimulation test results growth hormone peak > At 10 ng/mL, growth hormone was within the normal range.
3. no growth retardation caused by inherited metabolic diseases and various chronic diseases.

#### 2.1.2. Exclusion criteria

1. Children with chromosomal disorders, diabetes mellitus, thyroid dysfunction, significant infections, congenital heart disease, and other factors that affect growth and developmental indicators in this study.
2. Intracranial tumors or pituitary diseases by magnetic resonance examination before treatment; short stature caused by familial factors, central precocious puberty, hypothyroidism, Turner syndrome, multiple pituitary hormone deficiencies, minor for gestational age, malnutrition, and protein synthesis-related diseases.
3. Those with epiphyseal closure during treatment.
4. Those who did not use the medication regularly and follow up regularly.
5. Use of other drugs that affect the efficacy of the study drug.

### 2.2. Research methods

#### 2.2.1. rhGH treatment and follow-up

The dose of rhGH for children with GHD was 0.075 to 0.150 IU/(kg·d), and the amount for children with ISS was 0.125 to 0.200 IU/(kg·d). After treatment, the children were rechecked every three months. Data on children’s height, body mass index, changes in the development of secondary sex characteristics, and medication use were collected. Annual growth velocity and height standard deviation scores (HtSDS) were collected.

#### 2.2.2. Therapeutic efficacy evaluation indicators

Therapeutic efficacy evaluation indicators: Ht SDS was defined as(actual height of the child − standard standardized average height of children of the same age and sex) ÷ standard deviation of the height of children of the same age and sex; GV was defined as difference in height between two follow-up time points (cm) ÷ time interval (months) × 12, (cm/y); the changes in the height standard deviation scores (ΔHtSDS) as defined as(current HtSDS − HtSDS at last follow-up time point) ÷ time interval (months).

Pharmaco-economic evaluation index: into the calculation of the average cost-effectiveness ratio, an increase of 1 cm in height cost medical cost = (the cost of rhGH injections during the two follow-up visits + the cost of tests and examinations at the time of the follow-up visit) ÷ the difference in height between the two follow-up visits, (yuan/cm).

### 2.3. Statistical methods

Used SPSS version 27.0 to perform the statistical analysis. Measurement data conforming to normal distribution was expressed as $\bar{X}\pm S$. A paired *t* test was used to compare the means between the two groups and the median (interquartile range) for not conforming to the normal distribution, and a non-parametric test was used to compare the two groups. Count data were expressed as a rate (%) using the chi-square test. Differences were considered statistically significant at *P *< .05.

## 3. Results

### 3.1. Comparison of general characteristics of children with GHD and ISS before treatment

There was no statistically significant difference between the 2 groups regarding age, bone age, height, weight, BMI, and target height (*P *> .05). The 2 groups were comparable (Table 1).

**Table 1:** Clinical and biochemical characteristics of dwarf children.

	GHD	ISS	*P*
Number of cases	66	51	
Sex ratio, n (%)			
Male	40 (60.6)	28 (54.9)	.535
Female	26 (39.4)	23 (45.1)	
Age (yr)	10.92 (8.91–13.37)	10.83 (8.71–13.67)	.817
Bone age	10.00 (7.38–13.00)	10.00 (7.00–13.00)	.835
Height (cm)	129.63 ± 14.61	130.12 ± 16.21	.864
Weight (kg)	29.25 (23.50–38.69)	28.08 (22.00–38.18)	.562
BMI (kg/m^2^)	17.36 ± 2.52	16.76 ± 2.52	.205
GH peak (μg/L)	4.19 ± 2.14	14.18 ± 2.34	<.01
Target height (cm)			
Male	169.79 ± 3.69	169.85 ± 3.30	.947
Female	159.52 ± 3.87	157.93 ± 4.87	.215
Adolescent, n (%)			
Prepubescent	28 (42.4)	23 (45.1)	.772
Pubescent	38 (57.6)	28 (54.9)	

BMI = body mass index, GH peak = growth hormone peak, GHD = growth hormone deficiency, ISS = idiopathic short stature.

### 3.2. Comparison of the effect of rhGH in the treatment of children with GHD and ISS

**Figure 2.**
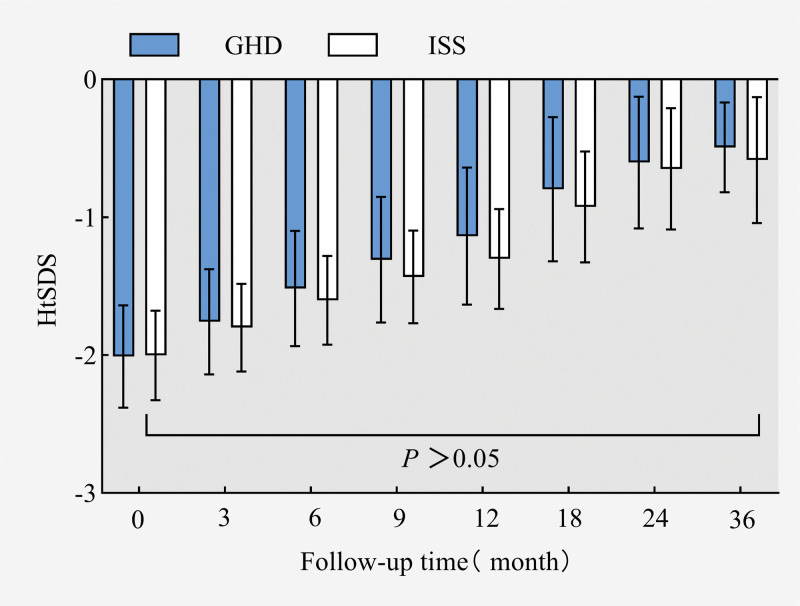
HtSDS changes after rhGH treatment. rhGH = recombinant human growth hormone, ΔHtSDS = the changes in the height standard deviation scores.

**Figure 3.**
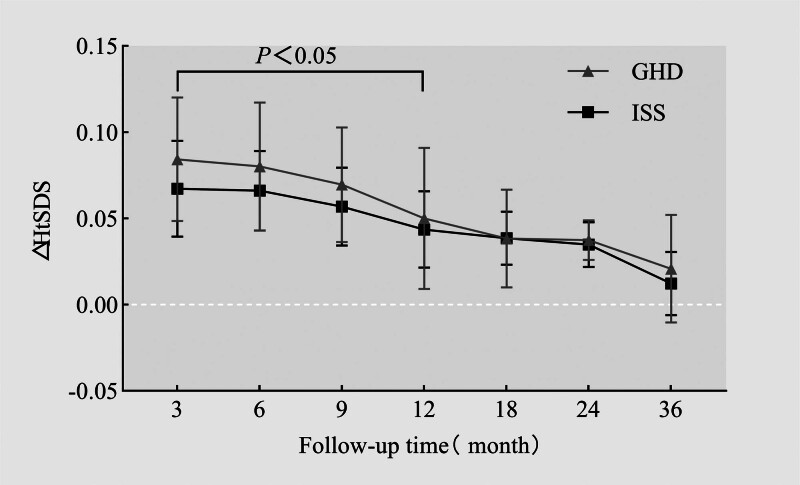
△HtSDS changes after rhGH treatment. rhGH = recombinant human growth hormone, ΔHtSDS = the changes in the height standard deviation scores.

**Figure 4.**
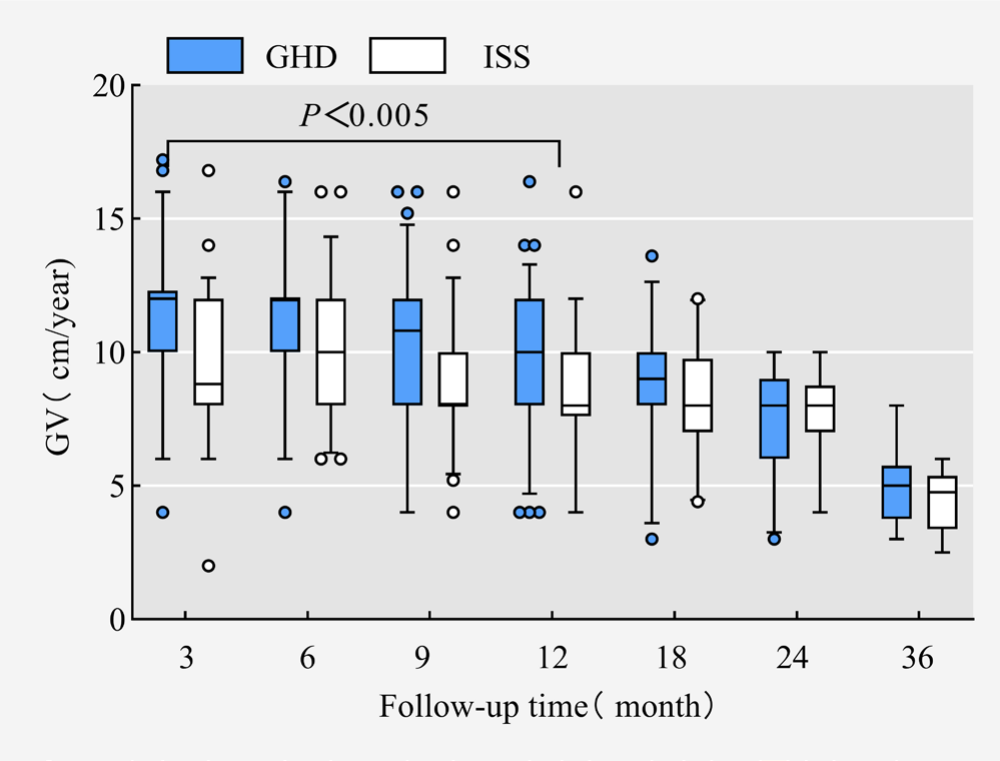
GV changes after rhGH treatment. GV = growth velocity, rhGH = recombinant human growth hormone.

After therapy with rhGH, the HtSDS of the GHD group was higher than that of the ISS group in all treatment periods, and with the prolongation of rhGH treatment time, the HtSDS of the two groups gradually increased and gradually tended to normalize to the expected average value of children of the same age and sex at 36 months of follow-up (Fig. 2); the HtSDS increased the fastest in 0 to 3 months after rhGH treatment and then slowed down with the prolongation of treatment time. The increase slowed down. The △HtSDS of the GHD group was higher than that of the ISS group at different time stages after treatment (*P* < .05 at months 3, 6, 9, and 12, the difference was statistically significant) (Fig. 3); after rhGH treatment, the GV of the children with GHD and ISS increased most significantly at the stage of months 0 to 6 and then slowed down gradually. The GV in the GHD group was higher than that in the ISS group at the 0 to 36 month stage after treatment (*P *< .05 at 3, 6, 9, and 12 months, statistically significant difference) (Fig. 4).

### 3.3. Economic cost analysis of rhGH treatment for ISS and GHD

GHD, ISS two groups within the respective pubertal stage group with the prolongation of the treatment time, every 1 cm height increase in the cost of medical expenses are increased compared with the same group of the previous treatment stage. GHD, ISS two groups of the respective treatment period of the pubertal children every 1cm height increase in the economic cost is higher than the same group of the pre-pubertal group within the same group, 3, 6, 9, 12, 18, 24 months intra-group comparisons (*P*_ab_, *P*_cd_ < .05) The difference was statistically significant. Inter-group comparison between the pre-pubertal groups of GHD and ISS (*P*_ac_ both > .05), inter-group comparison was not statistically significant, inter-group comparison between the pubertal groups of the GHD and ISS groups in March (*P*_bd_ = .033) and December (*P*_bd_ = .046) *P* < .05, the difference was statistically significant (Table 2).

**Table 2:** Medical costs for 1 cm height increase unit: yuan.

Time	GHD		ISS	
	Prepubescent (*n = 28)a	Pubescent (*n = 38)b	Prepubescent (*n = 23)c	Pubescent (*n = 28)d
3 mo	1597.52 (1048.87–2237.97)	2909.85 (2169.11–3572.19)	1840.77 (1618.75–2739.65)	3925.17 ± 1393.14
6 mo	1671.74 (1233.43–2303.64)	2865.89 (2244.92–3757.50)	2041.55 ± 562.07	3251.99 (2736.25–4132.98)
9 mo	1906.51 (1332.94–2961.38)	3270.38 (2661.49–4436.73)	2429.44 (1678.19–3092.86)	4356.48 ± 1546.28
12 mo	2346.00 ± 931.01	3554.71 (2798.53–4796.39)	2492.41 (1979.56–2749.38)	4464.68 (3334.47–6080.64)
18 mo	3342.37 ± 1002.79	3778.77 (3142.70–5417.13)	2919.67 ± 516.74	4980.58 (3788.85–5455.87)
24 mo	3778.77 (3023.02–4596.08)	4697.69 (4097.48–6807.46)	3199.58 ± 684.44	6369.10 ± 2362.8
36 mo	6665.21†	9252.70 ± 4007.31	5860.95 ± 1581.82	13277.75 ± 5781.41

GHD = growth hormone deficiency, ISS = idiopathic short stature.

* n represents the number of children within each pubertal subgroup at the initial time of treatment, abcd represents the GHD and ISS pubertal staging subgroups, respectively, and there was only 1 case in the pre-pubertal group of the

† GHD group that was followed up to 36 months.

## 4. Discussion

Since the US FDA first approved recombinant human growth hormone for clinical use in the treatment of dwarfism in 1985, rhGH has been widely used in the treatment of short stature with different etiologies. rhGH therapy is expensive, many scholars at home and abroad have highlighted the cost of rhGH therapy as an important issue for the healthcare system and society.^[5]^ In China, rhGH treatment for growth hormone deficiency was only included in medical insurance this year, but the reimbursement standard is strict, and most children still have to pay for growth hormone treatment at their own expense. The medical cost of rhGH treatment for short stature is also a concern for many families of children, and there are few studies on the economy of drug effect of short stature treatment. There are many causes leading to short stature, and a meta-analysis shows that GHD and ISS are the most representative study populations.^[6]^ There have been many studies on the efficacy of rhGH in the treatment of ISS in different populations.^[7]^ However, there are few reports on the differences in growth patterns between children with ISS treated with rhGH and patients with GHD. This study retrospectively analyzed the clinical data of 66 children with GHD and 51 children with ISS treated with rhGH for more than 1 year, and evaluated its efficacy and cost-effectiveness.

The results of this study showed that GV was higher in the first half year after rhGH treatment, and then decreased with the extension of treatment time. The study of Gou et al^[8]^ also found that GV was higher in the first half year of treatment, which was consistent with this study. The rapid increase in height during 3 to 6 months of rhGH treatment may be due to a catch-up growth phenomenon after the initial supplementation of deficient growth hormone.^[9]^ Consistent with the results of our study, Hou Ling et al^[10]^ showed that GV was higher in the GHD group than in the ISS group after rhGH treatment for half a year, this may indicate that the efficacy of GHD was better than that of ISS. After treatment, the HtSDS of GHD and ISS children were improved. The △HtSDS of the GHD group was higher than that of the ISS group. Al Shaikh et al^[11]^ showed that at the end of follow-up, HtSDS in GHD group and ISS group increased by 2.2 and 0.6 standard deviation score on average, respectively, and GHD children had a higher gain of HtSDS, which was consistent with the results of this study and Yoon et al.^[12]^ However, Schena et al^[13]^ found that after prepubertal children received rhGH treatment and were followed up until the children reached their final height, the HtSDS of the 2 groups increased in parallel, which may be related to the physical differences of different ethnic groups. GHD is a growth retardation caused by less rhGH secretion due to hypothalamic-anterior pituitary dysfunction, so rhGH supplementation for replacement therapy has a significant effect. However, the pathogenesis of ISS is complex,^[14]^ and the individual difference is great, and the effect of rhGH replacement therapy may not be as good as that of GHD children. Adult height difference between treated and untreated children with ISS is considered the primary efficacy outcome measure,^[15]^ and one study showed that a mean difference in height of more than 0.9 standard deviation score (about 6 cm) was considered a satisfactory response to rhGH treatment.^[16]^ Although arbitrary and flawed, this represents an acceptable cutoff for determining the minimum “ideal effect” of long-term rhGH therapy.

According to the National Health Insurance data of the United States, the annual medical cost of growth hormone treatment is US $28,805, and the cost of ISS children per 1 inch of height is US $52,634.^[5–17]^ In this study, the cost per 1 cm height gain of ISS children at different ages and treatment periods was higher than that of GHD children. Therefore, the cost-effectiveness of rhGH in children with GHD is higher than that in children with ISS. A meta-analysis by Pakdaman et al^[18]^ showed that children treated with rhGH grew more than 2.5 cm per year more than untreated children, at an average cost of US $20,000 per centimeter of height gain, and the Incremental cost effectiveness ratio varies in studies of various indications (ISS, GHD), The most cost-effective was GHD (from £20,000 to £30,000), which is consistent with the results of the study. However, the above study only included the drug costs of treatment and did not calculate all the costs of diagnosis and treatment. Furthermore, this meta-analysis distinguished between causes but not between ages. Our study included all the costs from diagnosis to follow-up and drug costs, and compared the cost-effectiveness of rhGH treatment in children with short stature at different ages and different treatment periods and different causes. The results showed that the cost of rhGH treatment per 1cm increase in height in the prepubertal group was lower than that in the adolescent group, and the longer the treatment time, the higher the economic burden. Previous studies have shown that the age of initiation of treatment is also one of the important factors of efficacy, and the cost of treatment is positively correlated with the time of initiation of treatment.^[19,20]^ Therefore, the earlier GH treatment is performed, the higher the overall benefit.

Our study still has some limitations, this study is not a prospective study, it only reports the follow-up observation results of 1 to 3 years of rhGH treatment, and the sample size is small. The medical records of children should be followed to further determine the efficacy of long-term rhGH treatment.

## 5. Conclusions

rhGH is effective in promoting height growth in children with dwarfism, with better results in children with GHD than in children with ISS; the earlier the treatment, the lower the medical costs and the higher the overall benefit.

## Author contributions

**Conceptualization:** Yanxia Ma, Lin Liu.

**Data curation:** Yanxia Ma, Jianping Sheng, Lijie Wang, Yanan Zhang.

**Investigation:** Yanxia Ma, Jianping Sheng.

**Methodology:** Yanxia Ma.

**Project administration:** Lin Liu.

**Writing – original draft:** Yanxia Ma.

**Writing – review & editing:** Yanxia Ma, Lin Liu.

## Supplementary Material


